# Assessment of Outcomes Associated With the Use of Newly Approved Oncology Drugs in Medicare Beneficiaries

**DOI:** 10.1001/jamanetworkopen.2021.0030

**Published:** 2021-02-24

**Authors:** Angela K. Green, Michael Curry, Niti Trivedi, Peter B. Bach, Sham Mailankody

**Affiliations:** 1 Gynecologic Medical Oncology Service, Department of Medicine, Memorial Sloan Kettering Cancer Center, New York, New York; 2 Center for Health Policy and Outcomes, Memorial Sloan Kettering Cancer Center, New York, New York; 3 Myeloma Service, Department of Medicine, Memorial Sloan Kettering Cancer Center, New York, New York

## Abstract

**Importance:**

A lack of generalizability of pivotal cancer clinical trial data to treatment of older adults with Medicare could affect therapeutic decision-making in clinical practice.

**Objective:**

To evaluate the differences in survival, duration of therapy, and treatment patterns between clinical trial patients and older adults with Medicare receiving cancer drugs for metastatic solid cancers in usual practice.

**Design, Setting, and Participants:**

This retrospective cohort study, performed from May 1, 2018, to August 30, 2020, used the linked Surveillance, Epidemiology, and End Results (SEER) program and Medicare database to examine sequential US Food and Drug Administration (FDA)–approved cancer drug indications (2008-2013) for locally advanced or metastatic solid tumors to assess whether pivotal trials reflect the outcomes of Medicare patients with cancer treated in usual practice.

**Exposures:**

Treatment with FDA-approved cancer drugs for metastatic solid cancers in pivotal clinical trials and in the SEER-Medicare database.

**Main Outcomes and Measures:**

Overall survival, duration of treatment, and dose reductions among trial participants and treated Medicare patients.

**Results:**

A total of 11 828 trial participants (mean age, 61.8 years; 6718 [56.8%] male; and 7605 [64.3%] White) and 9178 SEER-Medicare patients (mean age, 72.7 years; 4800 [52.3%] male; and 7437 [81.0% White]) were compared. Twenty-nine indications for 22 cancer drugs were included. Median overall survival among Medicare patients was shorter than among patients in the clinical trial intervention arm for 28 of 29 indications (median difference, −6.3 months; range, −28.7 to 2.7 months). Median duration of therapy among Medicare patients was shorter for 23 of the 27 indications with data available (median difference, −1.9 months; range, −12.4 to 1.4 months). For 9 indications, there was information available regarding dose reductions in the package insert or trial publication. In all but 1 instance, dose reductions or single prescriptions were more common in the Medicare population compared with dose reductions among the clinical trial patients; for example, in the Medicare patients, 600 of 1032 (58.1%) received dose reduction or a single prescription and 172 of 1032 (16.7%) received a single prescription vs 734 of 3416 (21.5%) in the trial intervention arm. The exception was afatinib for non–small cell lung cancer: 34 of 71 (47.9%) received dose reduction or a single prescription and 15 of 71 (21.1%) received a single prescription among the Medicare patients vs 120 of 230 (52.2%) receiving dose reductions among the trial intervention group.

**Conclusions and Relevance:**

In this cohort study, patients receiving Medicare who were treated with FDA-approved cancer drugs did not live as long as treated clinical trial participants and commonly received treatment modifications. This study suggests that cancer clinical data relevant to newly approved drugs lack generalizability to Medicare beneficiaries with cancer; therefore, these agents should be used with caution.

## Key Points

**Question:**

What are the real-world outcomes for Medicare patients with metastatic cancer receiving recently approved oncology drugs and how do they compare with pivotal clinical trial outcomes?

**Findings:**

In this retrospective cohort study, outcomes were compared between clinical trial participants and treated Medicare patients across 22 cancer drugs approved by the US Food and Drug Administration for 29 indications. Median duration of therapy and overall survival among treated Medicare patients were generally shorter than among trial participants, and dose reductions were common among Medicare patients.

**Meaning:**

Pivotal clinical trials may provide inadequate data for the purpose of clinical decision-making among Medicare beneficiaries with advanced cancer.

## Teaser

This cohort study evaluates the differences in survival, duration of therapy, and treatment patterns between clinical trial patients and older adults with Medicare receiving cancer drugs for metastatic solid cancers in usual practice.

## Introduction

Differences between clinical trial participants and the broader patient population they are intended to represent can impair the generalizability of study results.^1,2,3,4,5,6^ Elderly, minority, poor, and chronically ill patients, for instance, are often underrepresented in cancer clinical trials.^7,8,9,10,11,12^ Lack of generalizability of clinical trials for cancer drugs used to treat patients with metastatic cancers may be of particular concern. Such therapies tend to have modest efficacy and produce substantial toxic effects even in young, fit clinical trial participants. Thus, additional scrutiny is warranted when applying these trial data to patients at a higher risk of harm from therapy.^13,14^

Adults 65 years and older are projected to account for 70% of cancer diagnoses by 2030. Medicare, the US federal health insurance program for elderly and disabled people, provides coverage for more than 50 million older adults, including nearly 7.5 million with cancer. In 2018, more than 40% of prescription dispensations for oral cancer drugs used to treat metastatic cancers were to Medicare beneficiaries.^15^ However, trial representation of patients in this age group is routinely lower, and age disparities between trial participants and the incident disease population is widening.^16,17,18^

Adequate power for subgroup analyses is needed for clinical trial results to be generalizable. Although this is not the same as representativeness—the extent to which a clinical trial cohort reflects the underlying disease population—improving the representation of older patients could increase the sample size sufficiently to perform these subgroup analyses needed for scientific inference. Given that older patients may experience more toxic effects when receiving cancer treatments, inadequate generalizability may be of clinical consequence.^19^

Prior research^20^ suggests that outcomes of cancer treatment differ between clinical studies and patients treated in usual clinical practice. Sanoff et al^21^ found that patients receiving sorafenib for advanced hepatocellular carcinoma had shorter survival than their clinical trial counterparts (median survival, 3 vs 10.3 months). Khozin et al^22^ also found a survival deficit among patients with metastatic non–small cell lung cancer (NSCLC) treated with nivolumab or pembrolizumab (median survival, 8 vs 9.2-12.2 months). Schmidinger et al,^23^ however, found that patients receiving pazopanib for advanced renal cell carcinoma in everyday practice lived longer than individuals in the pivotal clinical trial (median, 29.9 vs 22.9 months).

Using the Surveillance, Epidemiology, and End Results (SEER)–Medicare database, we conducted a study aimed to examine the generalizability of pivotal cancer clinical trial data. First, we compared the survival of Medicare patients treated with new drugs with that among pivotal clinical trial participants. Second, we examined whether the duration of treatment differed between Medicare patients and pivotal clinical trial participants. Third, we assessed the frequency of early discontinuation of drug use and dose reductions among Medicare patients treated with new drugs. We did not assess the incremental survival benefit of newly approved cancer drugs among Medicare patients.

## Methods

This retrospective cohort study was conducted from May 1, 2018, to August 30, 2020, using the linked SEER-Medicare database. The study followed the Strengthening the Reporting of Observational Studies in Epidemiology (STROBE) reporting guideline. Given that the SEER database contains deidentified data, informed consent was not possible, and the study was approved as exempt research by the institutional review board at Memorial Sloan Kettering Cancer Center.

### Approved Cancer Drug Indications

We abstracted sequential cancer drug approvals for metastatic and locally advanced indications without curative intent approved between January 1, 2008, and December 31, 2013, from the US Food and Drug Administration (FDA) Hematology/Oncology (Cancer) Approvals and Safety Notifications website^24^ (eFigure 1 in the Supplement). We only included drugs for which median overall survival in the intervention arm of the pivotal trial, defined as the trial cited on the package insert (the label) and the FDA approval announcement, was published in a peer-reviewed journal. Median duration of therapy, dose reductions, and age-specific survival results were recorded from the trial publication or FDA package insert when available.

### Medicare Patient Outcomes

We identified patients in the SEER-Medicare database diagnosed with cancer between January 1, 2006, and December 31, 2015, whose disease type and stage matched the FDA indication and who received at least 1 dose of the relevant cancer drug via intravenous infusion or filled 1 prescription for oral therapy (eTable 1 in the Supplement). We did not further limit the patient population to those who met trial eligibility criteria because our goal was to study those treated in general. We assumed the presence of biomarkers when relevant because SEER does not routinely capture such information. SEER is a population-based cancer registry that covers 28% of the US population. Medicare claims capture health care use and date of death.^25^

Patients had stage IV disease at diagnosis unless the indication included patients with locally advanced disease not amenable to curative surgery or radiotherapy. In such cases, patients with stage III disease at diagnosis were included if they had no cancer-directed surgery and/or radiotherapy documented in the SEER-Medicare database. Treatment was determined from J codes in Medicare Part B for intravenous drugs and generic and brand names in Medicare Part D for oral drugs. Median duration of therapy was measured from the date of first fill to the end of the days supplied of the last fill for oral drugs and from the first to last date of drug administration, unless the trial duration of therapy was reported in cycles, for the intravenous drugs. If so, we approximated number of cycles in the SEER-Medicare database and converted to months of therapy for more accurate comparison (eTable 2 in the Supplement). We restricted the sample to patients with continuous enrollment in Medicare Parts A and B for 6 months before and after diagnosis or until death or end of follow-up. More than 99% of patients receiving oral therapy in our analysis had continuous Medicare Part D enrollment.

### Statistical Analysis

We included drug indications for which 30 or more SEER-Medicare patients met eligibility criteria. We compared age by regressing the mean age of patients in the SEER-Medicare database (weighted by sample size) against the mean age in the intervention arm of the pivotal trial because variance was not available for the trial population. A test of proportions was used to examine significant differences in race/ethnicity and sex composition. Statistical significance was defined as a 2-sided *P* ≤ .05.

Kaplan-Meier survival analyses were used to calculate median overall survival (censoring date December 31, 2016) and cancer-specific survival (censoring date December 31, 2015, the follow-up duration available in SEER-Medicare for this outcome) for SEER-Medicare patients. The burden of comorbidities for SEER-Medicare patients treated for each drug indication was calculated using the Charlson comorbidity index, modified to exclude cancer diagnoses, and categorized into 3 groups according to number of comorbidities (0-1, 2, and ≥3).^26,27^ For each drug indication, we calculated the absolute and relative differences between median overall survival and duration of therapy and summarized these differences by the median and range of values across indications. Overall survival estimates were also calculated among the subgroup of Medicare patients with 1 or no comorbidities (Charlson comorbidity index, 0-1).

If a prescription for an oral drug was filled for less than any of the approved doses for that indication and there was no later claim for a recommended dose, this was counted as a dose reduction. It is not possible to determine whether intravenous doses match those approved by the FDA.^28^ When a patient received only 1 prescription claim (for 30-90 days) or a single cycle of an intravenous drug, we labeled this a single treatment.

## Results

A total of 11 828 trial participants (mean age, 61.8 years; 6718 [56.8%] male; 7605 [64.3%] White) and 9178 SEER-Medicare patients (mean age, 72.7 years; 4800 [52.3%] male; 7437 [81.0%] White) were compared. The median difference in age across indications was 11 years, with a range of 1 to 20 years. A total of 8031 treated SEER-Medicare patients (87.5%) across indications had 1 or no comorbidities.

A total of 47 drug indications across 33 drugs were approved by the FDA for metastatic or locally advanced, noncurative solid tumor treatment between 2008 and 2013 (eFigure 1 in the Supplement). Overall survival data for the intervention arm of the pivotal trial were available for 38 indications that represented 27 drugs. Of these, there were sufficient numbers of patients in SEER-Medicare to evaluate outcomes for 29 indications across 22 drugs (Table 1).

**Table 1.** Drug Indications Included in Analysis With Baseline Demographics Among SEER-Medicare Patients and Clinical Trial Intervention Arm

Year of FDA approval	Drug	FDA indication^a^	SEER-Medicare/trial intervention arm				Modified Charlson comorbidity index, (range, 0-1), No. (%)
			Sample size	Age, median (IQR)/ y	No. (%)		
					White race	Male sex	
2008	Pemetrexed	NSCLC, first line	720/862	70 (67-73)/61	605 (84.0)/672 (78.0)	382 (53.1)/603 (70.0)	653 (90.7)
2009	Everolimus	Kidney cancer	105/272	70 (66-75)/61	84 (80.0)/NR	64 (61.0)/212 (77.9)	86 (81.9)
2009	Pazopanib	Kidney cancer	295/290	72 (67-78)/59	245 (83.0)/252 (86.9)	189 (64.1)/197 (67.9)	235 (79.7)
2009	Pemetrexed	NSCLC, maintenance	2192/359	73 (69-77)/61	1885 (86.0)/337 (93.9)	1096 (50.0)/201 (56.0)	1901 (86.7)
2010	Erlotinib	NSCLC, maintenance	208/438	71 (67-76)/60	156 (75.0)/368 (84.0)	114 (54.8)/320 (73.1)	165 (79.3)
2010	Cabazitaxel	Prostate cancer	220/378	71 (68-76)/68	187 (85.0)/325 (86.0)	220 (100.0)/378 (100.0)	207 (94.1)
2010	Eribulin	Breast cancer	43/508	68 (66-73)/55	36 (83.7)/472 (92.9)	0/0	41 (95.3)
2010	Trastuzumab	Gastric cancer	43/294	71 (66-74)/59	36 (83.7)/115 (39.1)	32 (74.4)/226 (76.9)	38 (88.3)
2010	Sipuleucel-T	Prostate cancer	331/341	73 (69-79)/72	285 (86.1)/303 (88.5)	331 (100.0)/341 (100.0)	290 (87.6)
2011	Everolimus	Pancreatic NET	53/207	72 (68-76)/58	47 (88.7)/155 (74.9)	30 (56.6)/110 (53.1)	45 (84.9)
2011	Abiraterone	Prostate cancer, prior docetaxel	122/797	72 (68-75)/69	78.7 (96.0)/NR	122 (100.0)/797 (100.0)	115 (94.3)
2011	Vemurafenib	Melanoma	84/337	70 (67-77)/56	46 (97.6)/199 (99.1)	82 (54.8)/334 (59.1)	75 (89.3)
2011	Cetuximab	Head and neck cancer	55/222	68 (67-74)/56	87.3 (48)/NR	83.4 (46)/89.2 (198)	46 (83.6)
2011	Ipilimumab	Melanoma	32/137	73 (68-78)/56	32 (100.0)/129 (94.2)	22 (68.8)/84 (61.3)	26 (81.3)
2012	Everolimus	Breast cancer	97/485	71 (68-77)/62	79 (81.4)/359 (74.0)	0/0	91 (93.8)
2012	Axitinib	Kidney cancer	88/361	71 (67-76)/61	74 (84.0)/278 (77.0)	60 (68.2)/264 (73.1)	75 (85.2)
2012	Nab-paclitaxel	NSCLC	179/521	75 (68-80)/60	141 (78.8)/417 (80.0)	104 (58.1)/391 (75.0)	145 (81.0)
2012	Enzalutamide	Prostate cancer	215/800	72 (68-76)/69	215 (78.1)/800 (93.0)	215 (100.0)/800 (100.0)	192 (89.3)
2012	Abiraterone	Prostate cancer	742/546	74 (69-80)/71	556 (74.9)/NR	742 (100.0)/546 (100.0)	621 (83.7)
2012	Regorafenib	Colorectal cancer	295/505	70 (66-74)/61	224 (75.9)/394 (78.0)	162 (54.9)/313 (61.9)	270 (91.5)
2012	Cetuximab	Colorectal cancer	280/316	70 (67-74)/61	227 (81.1)/NR	174 (62.1)/196 (62.0)	248 (88.6)
2012	Pertuzumab	Breast cancer	88/402	70 (66-76)/54	67 (76.1)/245 (60.9)	0/0	76 (86.4)
2012	Ziv-aflibercept	Colorectal cancer	79/612	73 (68-76)/61	43 (67.1)/367 (90.0)	53 (54.4)/551 (60.0)	71 (89.9)
2013	Trastuzumab emtansine	Breast cancer	53/495	71 (66-77)/53	43 (81.1)/356 (71.9)	0 (0)/1 (0.2)	48 (90.6)
2013	Dabrafenib	Melanoma	51/187	73 (67-79)/53	50 (98.0)/187 (100)	30 (58.8)/112 (59.9)	44 (86.3)
2013	Nab-paclitaxel	Pancreatic cancer	1019/431	73 (68-77)/62	866 (85.0)/379 (87.9)	530 (52.0)/246 (57.1)	853 (83.7)
2013	Afatinib	NSCLC	71/230	77 (71-84)/62	51 (71.8)/62 (27.0)	19 (26.8)/83 (36.1)	64 (90.1)
2013	Erlotinib	NSCLC	1032/86	77 (67-76)/65	65.0/90.7	299 (29.0)/28 (32.6)	866 (83.9)
2013	Bevacizumab	Colorectal cancer	386/409	71 (67-75)/63	305 (79.0)/NR	216 (56.0)/266 (65.0)	352 (91.2)

Abbreviations: FDA, US Food and Drug Administration; IQR, interquartile range; nab, nanoparticle albumin-bound; NET, neuroendocrine tumor; NR, not reported in clinical trial publication, FDA package insert, or ClinicalTrials.gov; NSCLC, non–small cell lung cancer; SEER, Surveillance, Epidemiology, and End Results.

^a^ Complete FDA-approved indications are listed in eTable 1 in the Supplement.

**Figure 1.**
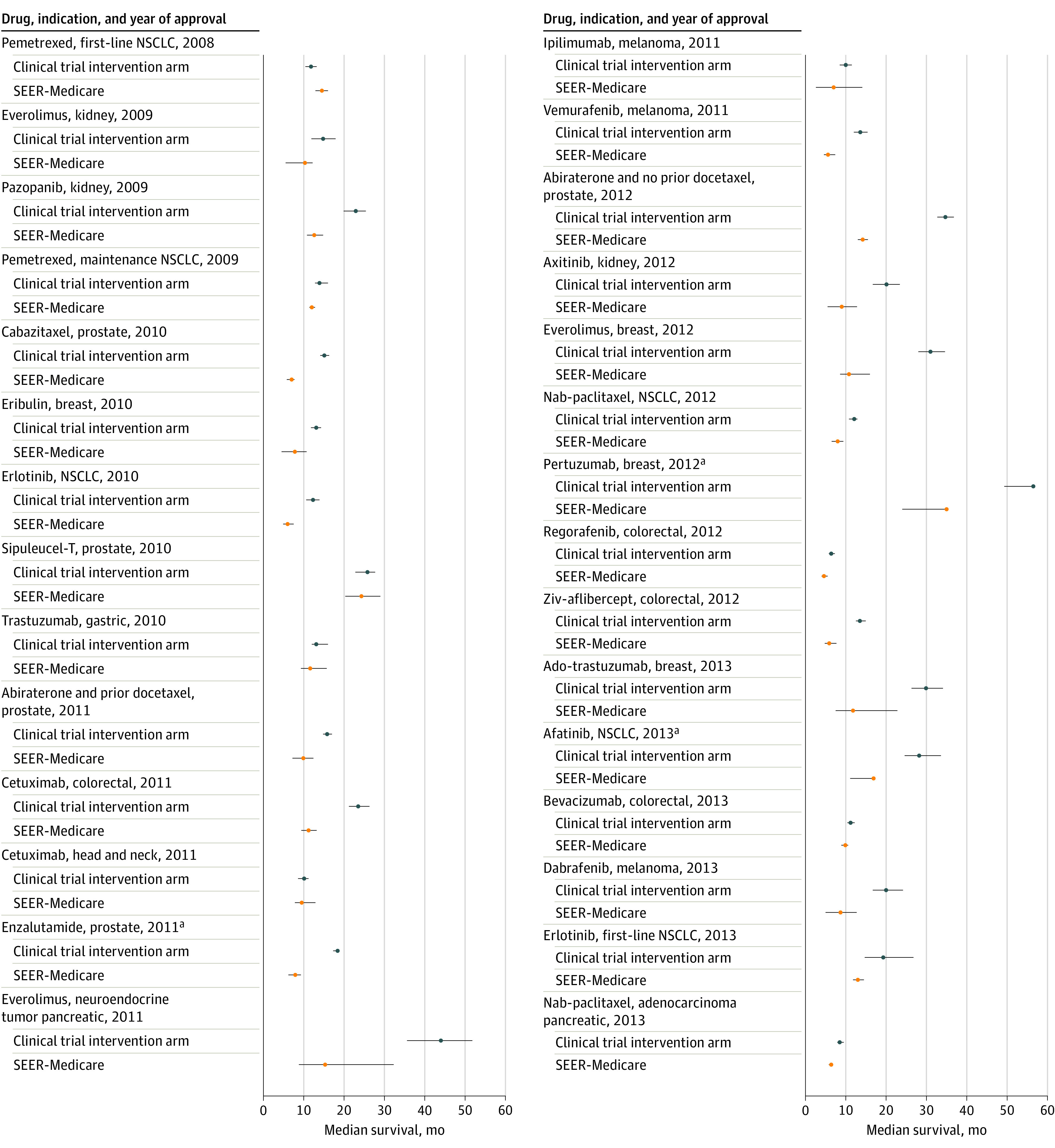
Median Overall Survival (95% CIs) Comparing Surveillance, Epidemiology, and End Results (SEER)–Medicare Patients and Clinical Trial Intervention Arm Participants Receiving the Same US Food and Drug Administration–Approved Cancer Drug for the Same Indication (2008-2013) Nab indicates nanoparticle albumin-bound; NSCLC, non–small cell lung cancer. ^a^Upper confidence bound not met.

For 28 of the 29 drug indications (22 drugs), the median survival among treated SEER-Medicare patients was shorter than among clinical trial participants receiving the same treatment for the same indication (14.0 vs 11.8 months) (Figure 1). The exception was pemetrexed for the first-line treatment of NSCLC. The median survival of Medicare patients was 59.9% of the survival of clinical trial patients, corresponding to a difference of 6.3 months at the median (range, −28.7 to 2.7 months). For 12 indications (41.4%), the median survival of SEER-Medicare patients was less than half the duration observed among patients in the clinical trial (median absolute difference, −11.2 months; range, −28.7 to −6.3 months). The median survival of Medicare patients with 1 or no comorbidities was 59.5% of the survival of clinical trial patients (median absolute difference, −6.7 months; range, −24.4 to 2.7 months) (eFigure 2 in the Supplement).

In SEER-Medicare (eTable 3 in the Supplement) across all indications, 8141 patient deaths (88.7%) were attributed to cancer (range, 78.5% to 100.0%). Only 6 of the 29 indications (5 drugs) reported age-specific survival in the trial publication or FDA package insert (eTable 4 in the Supplement). The median overall survival for older trial participants was similar to that for younger trial participants and longer than survival among patients in SEER-Medicare for 5 indications (4 drugs). It was similar for patients receiving cetuximab for head and neck cancer (median survival in trial among those ≥65 years of age, 9.1 months; median survival in SEER-Medicare group, 9.7 months).

**Figure 2.**
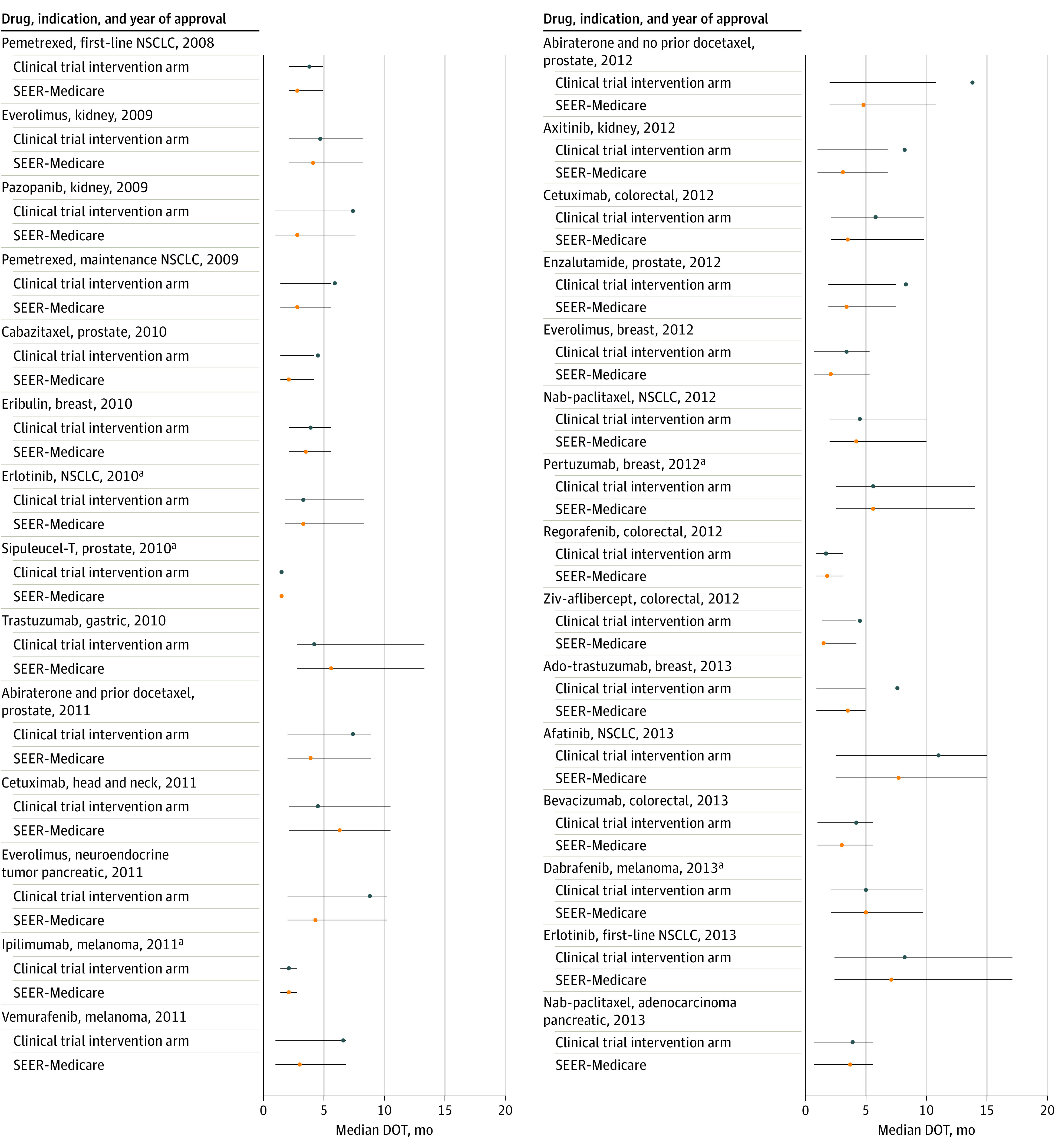
Median Duration of Therapy (DOT) (Interquartile Ranges) Comparing Surveillance, Epidemiology, and End Results (SEER)–Medicare Patients and Clinical Trial Intervention Arm Participants Receiving the Same US Food and Drug Administration–Approved Cancer Drug for the Same Indication (2008-2013) Nab indicates nanoparticle albumin-bound; NSCLC, non–small cell lung cancer. ^a^Upper confidence bound not met.

There were 27 indications (21 drugs) for which median duration of therapy was available for the clinical trial participants in the trial publication or FDA package insert for comparison with Medicare beneficiaries, and it was shorter for Medicare beneficiaries in 23 of them (Figure 2). Median duration of therapy among Medicare patients was 59.0% of treatment duration for clinical trial patients across indications, for a difference of 1.9 months at the median (range, −12.4 to 1.4 months). Across all indications, 1725 SEER-Medicare patients (18.8%) received a single prescription or cycle, ranging from 1 patient (2.3%) (trastuzumab for gastric cancer) to 120 patients (40.7%) (regorafenib for colorectal cancer). Among Medicare patients, 1459 (42.2%) were dose reduced, ranging from 20 (16.4%) to 64 (66.0%) (Table 2) across the 14 oral drug indications (10 drugs) for which this outcome could be evaluated. For 9 of these indications, information was available regarding dose reductions in the package insert or trial publication. In all but 1 instance (afatinib for NSCLC: 34 of 71 [47.9%] receiving dose reduction or a single prescription and 15 of 71 [21.1%] receiving a single prescription in the Medicare patients vs 120 of 230 [52.2%] receiving dose reductions in the trial intervention arm), dose reductions or single prescriptions were more common in the Medicare population compared with dose reductions among the clinical trial patients (eg, 600 of 1032 [58.1%] receiving dose reduction or a single prescription and 172 of 1032 [16.7%] receiving a single prescription in the Medicare patients vs 734 of 3416 [21.5%] in the trial intervention arm) (Table 2). For erlotinib, 18 of 84 patients (21.4%) with first-time NSCLC had dose reduction.

**Table 2.** Dose Reductions or Single Prescriptions Among SEER-Medicare Patients Who Received Oral Medicare Part D Drugs Compared With Dose Reductions in the Clinical Trial Intervention Arm

Drug, indication	SEER-Medicare arm, No./total No. (%)		Dose reductions in trial intervention arm, No./total No. (%)
	Dose reduction or single prescription	Single prescription	
Everolimus, breast	64/97 (66.0)	22/97 (22.7)	NR
Axitinib, RCC	37/88 (42.0)	24/88 (27.3)	121/359 (34.0)
Everolimus, RCC	32/105 (30.5)	19/105 (18.1)	NR
Pazopanib, RCC	154/295 (52.2)	89/295 (30.2)	104/290 (36.0)
Erlotinib, NSCLC maintenance	100/208 (48.2)	43/208 (20.7)	70/433 (16.0)
Everolimus, pancreatic NET	26/53 (49.1)	10/53 (18.9)	NR
Enzalutamide, CRPC	51/215 (23.7)	45/215 (20.9)	NR
Abiraterone, CRPC	132/742 (17.8)	111/742 (15.0)	38/542 (7.0)
Abiraterone, CRPC with prior docetaxel	20/122 (16.4)	18/122 (14.8)	23/791 (2.9)
Regorafenib, colorectal	159/295 (53.9)	120/295 (40.7)	188/500 (38.0)
Vemurafenib, melanoma	37/84 (44.0)	32/84 (38.1)	NR
Afatinib, NSCLC	34/71 (47.9)	15/71 (21.1)	120/230 (52.0)
Dabrafenib, melanoma	19/51 (37.3)	11/51 (21.6)	52/187 (28.0)
Erlotinib, NSCLC first line	600/1032 (58.2)	172/1032 (16.7)	18/84 (21.4)

Abbreviations: CRPC, castration-resistant prostate cancer; NET, neuroendocrine tumor; NR, not reported in clinical trial publication, FDA package insert, or ClinicalTrials.gov; NSCLC, non–small cell lung cancer; RCC, renal cell carcinoma; SEER, Surveillance, Epidemiology, and End Results.

## Discussion

This cohort study was conducted to evaluate the generalizability of pivotal trial data to Medicare beneficiaries treated in usual clinical practice. The median overall survival, duration of therapy, and treatment patterns were compared between Medicare patients treated with FDA-approved cancer drugs and the participants in the pivotal trials. With only 1 exception (afatanib), the median survival across drugs and indications was briefer for the Medicare patients, with a median absolute difference in survival of 6.3 months. Medicare patients received shorter durations of therapy than study participants, and sizeable percentages received only a single prescription or cycle (18.8%) or dose reductions (42.2%). The effectiveness or incremental survival benefit cannot be determined from these new treatments for Medicare patients because of the lack of a comparison group of patients who did not receive treatment and because this was not the aim of the study.

Several associated factors are likely to explain these differences. In the analysis, the mean age of Medicare patients receiving treatment exceeded the mean age of clinical trial participants by 11 years (73 vs 62 years). Advanced age is on average associated with frailty and a higher prevalence of comorbidities, which may heighten drug toxicity and reduce patients’ ability to tolerate cancer drugs.^29^ These factors may lead to earlier discontinuation of therapy, treatment interruptions, or suboptimal dosages, negatively impacting survival. Although this study found that Medicare beneficiaries received shorter durations of therapy than study participants, including a sizeable percentage receiving only a single prescription or cycle as well as dose reductions, this was likely not solely attributable to their age but also to other factors, including comorbidities, performance status, tumor prognostic factors, access to care, racial disparities, systemic discrimination, and other socioeconomic factors that could lead to differential treatment and outcomes in the Medicare population compared with clinical trial participants.

In the few trials in which age-specific survival was reported in the publication or FDA package insert, survival was generally similar between older and younger study participants, suggesting that age alone does not explain our findings. However, this result was only available in 6 (20.7%) of the pivotal trials included. Another reason why advanced age might be associated with poorer outcomes is that older patients die at higher rates from other causes. However, this study found that nearly 90% of deaths among SEER-Medicare patients were associated with cancer.

Beyond older age, comorbidities or illness from more advanced or aggressive cancer that impairs drug tolerability could explain why, among Medicare patients, therapies were routinely discontinued early, doses were reduced, or medication was given for a single prescription or cycle of therapy. Most patients in the Medicare cohort had mild or no comorbidities (87.5%), and only small changes in survival were noted when the analysis was limited to this subgroup. However, the modified Charlson comorbidity index, a weighted score based on chronic disease diagnostic codes, may not be associated with the typical measures of performance status in patients with advanced cancer, such as the Eastern Cooperative Oncology Group and Karnofsky performance status scores, which are not available in the SEER-Medicare database. Furthermore, intrinsic delays in the enrollment process can lead investigators to enroll trial patients with less aggressive disease and thus better prognosis. Patients are often excluded from trials if they are heavily pretreated or their disease is rapidly progressing. In usual practice, patients may receive approved drugs after other prior treatments, when they are sicker or closer to the end of life.

This study found that the median survival and duration of treatment of patients in cancer clinical trials was substantially longer than that of treated Medicare patients. Although possibly expected for reasons outlined, these findings suggest that pivotal trial data do not reflect the experience of Medicare patients treated in usual clinical practice. As a result, the risks vs benefits of treatment are difficult to surmise. In particular, understanding the influence of dose reductions and prescribing patterns on survival outcomes in Medicare beneficiaries is an area for further study. Avoiding subtherapeutic dosages without clear indication for dose reduction may be a modifiable intervention to improve survival in older Medicare patients with cancer; however, the optimal dosing strategy in older adults is poorly defined.^30^

This study may have implications for clinical trial design to improve the external validity of pivotal trials and regulatory decision-making to ensure that data for clinical prescribing in Medicare patients is informative. Broadening eligibility criteria might improve but cannot ensure the sample size required for generalizability of cancer clinical trial data.^31^ Allowances for a wider range of performance status, preexisting comorbidities, and organ dysfunction, however, would improve the characterization of toxic effects of treatment. The FDA released guidance to encourage researchers and sponsors to broaden eligibility criteria^32^; whether these changes are implemented remains to be seen.

The FDA could encourage pharmaceutical firms to design their pivotal trials in a manner that ensures availability of age-specific toxic effects, dosing, and efficacy data, which were inconsistently available in the published literature and often nonspecific in the FDA package inserts. The Institute of Medicine has suggested patent extensions for companies that pursue dedicated trials in older patients or those with comorbidities. These extensions are important for oncologic drugs because more than 40% of prescriptions in 2018 for oral cancer drugs included in this analysis are administered to Medicare patients.^15^

Real-world data can be used to generate additional information to help guide treatment decisions in understudied patients and could also be required by the FDA as part of postmarketing commitments. However, postapproval trials and mandatory surveillance studies are often underresourced and inconsistently performed.^33,34,35^ Regardless, evidence of poorer outcomes or increased toxic effects in specific subgroups could lead to a requirement for more formal phase 4 studies.

### Limitations

This study has limitations. An important limitation is the lack of a comparator group of Medicare patients who did not receive the treatment being evaluated; therefore, any estimate of the relative survival benefit from these drugs among Medicare patients could not be determined. The generalizability of clinical trial outcomes to younger, non-Medicare patients could also not be assessed, and similar differences may be evident among these patients. Because of limitations of claims-based data, the role of such factors as age, comorbidities, and socioeconomic and demographic variables in the observed differences in clinical trial efficacy in Medicare patients cannot be quantified. This analysis was limited to patients with stage IV or noncurative stage III cancer at diagnosis because these patients can be reliably identified in the SEER database. Many of the pivotal trials also include participants with newly metastatic disease; however, this was unlikely to influence the study findings because the difference in prognosis among patients with recurrence vs stage IV disease at diagnosis is mixed,^36,37^ and stage IV diagnosis at presentation is common for cancers included in this analysis.^38,39,40,41^

## Conclusions

Among Medicare patients with advanced solid cancers treated with FDA-approved drugs, median survival was shorter than that reported among clinical trial participants treated with the same drugs for all but 1 drug used for 1 indication. Many Medicare patients were also treated for only brief duration, had dose reductions, or received a single prescription or treatment cycle. These findings raise concerns regarding the generalizability of clinical trial data for treatment decision-making in Medicare patients. Furthermore, prescribing patterns in Medicare patients require additional scrutiny to ensure optimal dosing to avoid overtreatment or undertreatment in this population. Pivotal trials can be improved, and regulatory requirements could emphasize the importance of generating data relevant to the older patients who constitute an increasing number of all patients with cancer in the US.^11^

## Notes

Supplement. **eFigure 1.** Flow Diagram of Selection of Cancer Drug Indications **eFigure 2.** Median Overall Survival With 95% Confidence Intervals Comparing SEER-Medicare Patients With Charlson Comorbidity Index 0-1 and the Clinical Trial Intervention Arm Participants Receiving the Same FDA Approved Cancer Drug for the Same Indication **eTable 1.** SEER-Medicare Inclusion Criteria for Cohort Selection **eTable 2.** Cycle Estimates for Intravenous Cancer Drugs Used to Calculate Duration of Therapy and/or Single Cycles **eTable 3.** Median Cancer-Specific Survival and Percent Deaths Due to Cancer Among SEER-Medicare Patients, Censored 12/31/2015 **eTable 4.** Age-Specific Median Overall Survival or Hazard Ratios for Clinical Trial Intervention Arms
