# Supplementary material for: Assessment of Outcomes Associated With the Use of Newly Approved Oncology Drugs in Medicare Beneficiaries
Source: JAMA Netw Open. 2021 Feb 24;4(2):e210030. doi: 10.1001/jamanetworkopen.2021.0030 (PMC7905499; doi:10.1001/jamanetworkopen.2021.0030)
Supplement: Supplement. — eFigure 1. Flow Diagram of Selection of Cancer Drug Indications eFigure 2. Median Overall Survival With 95% Confidence Intervals Comparing SEER-Medicare Patients With Charlson Comorbidity Index 0-1 and the Clinical Trial Intervention Arm Participants Receiving the Same FDA Approved Cancer Drug for the Same Indication eTable 1. SEER-Medicare Inclusion Criteria for Cohort Selection eTable 2. Cycle Estimates for Intravenous Cancer Drugs Used to Calculate Duration of Therapy and/or Single Cycles eTable 3. Median Cancer-Specific Survival and Percent Deaths Due to Cancer Among SEER-Medicare Patients, Censored 12/31/2015 eTable 4. Age-Specific Median Overall Survival or Hazard Ratios for Clinical Trial Intervention Arms [file jamanetwopen-e210030-s001.pdf]

Supplementary Online Content

Green AK, Curry M, Trivedi N, Bach PB, Mailankody S. Assessment of outcomes associated with the use of newly approved oncology drugs in Medicare beneficiaries. *JAMA Netw Open*. 2021;4(2):e210030. doi:10.1001/jamanetworkopen.2021.0030

eFigure 1. Flow Diagram of Selection of Cancer Drug Indications
eFigure 2. Median Overall Survival With 95% Confidence Intervals Comparing SEER-Medicare Patients With Charlson Comorbidity Index 0-1 and the Clinical Trial Intervention Arm Participants Receiving the Same FDA Approved Cancer Drug for the Same Indication
eTable 1. SEER-Medicare Inclusion Criteria for Cohort Selection
eTable 2. Cycle Estimates for Intravenous Cancer Drugs Used to Calculate Duration of Therapy and/or Single Cycles
eTable 3. Median Cancer-Specific Survival and Percent Deaths Due to Cancer Among SEER-Medicare Patients, Censored 12/31/2015
eTable 4. Age-Specific Median Overall Survival or Hazard Ratios for Clinical Trial Intervention Arms

This supplementary material has been provided by the authors to give readers additional information about their work.

© 2021 Green AK et al. *JAMA Network Open*.

eFigure 1. Flow Diagram of Selection of Cancer Drug Indications

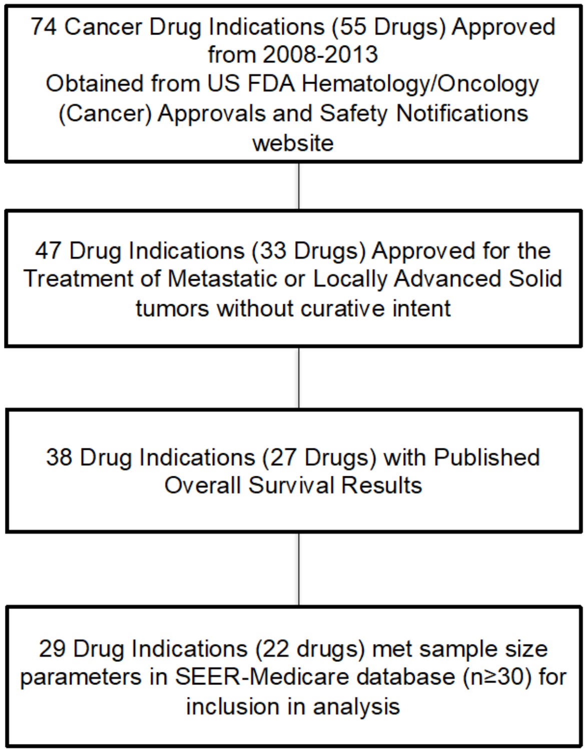

© 2021 Green AK et al. *JAMA Network Open.*

eFigure 2. Median Overall Survival With 95% Confidence Intervals Comparing SEER-Medicare Patients With Charlson Comorbidity Index 0-1 and the Clinical Trial Intervention Arm Participants Receiving the Same FDA Approved Cancer Drug for the Same Indication

	Survival Difference	
	Absolute (months)	Relative (%)
Pemetrexed, First Line NSCLC, 2008	2.7	122.9
Everolimus Kidney, 2009	−4.5	69.6
Pazopanib Kidney, 2009	−8.3	63.8
Pemetrexed, Maintenance NSCLC, 2009	−1.7	87.8
Cabazitaxel Prostate, 2010	−8.2	45.7
Eribulin Breast, 2010	−5.3	59.5
Erlotinib NSCLC, 2010	−6.7	45.5
Sipuleucel−T Prostate, 2010	0.4	101.6
Trastuzumab Gastric, 2010	−0.7	94.7
Abiraterone, Prior Docetaxel Prostate, 2011	−4.7	70.3
Cetuximab Head and Neck, 2011	0	100
Everolimus, Neuroendocrine Tumor Pancreatic , 2011	−24.4	44.5
Ipilimumab Melanoma, 2011	−4.4	56
Vemurafenib Melanoma, 2011	−7.8	42.6
Abiraterone, No Prior Docetaxel Prostate, 2012	−19.3	44.4
Axitinib Kidney, 2012	−10.9	45.8
Cetuximab Colorectal, 2012	−12.2	48.1
Enzalutamide Prostate, 2012*	−10.3	44
Everolimus Breast, 2012	−20	35.5
Nab−Paclitaxel NSCLC, 2012	−3.7	69.4
Pertuzumab Breast, 2012*	−21.8	61.4
Regorafenib Colorectal, 2012	−1.4	78.1
Ziv−Aflibercept Colorectal, 2012	−7.6	43.7
Ado−trastuzumab Breast, 2013*	−18.1	39.5
Afatinib NSCLC, 2013*	−11.9	57.8
Bevacizumab Colorectal, 2013	−1.5	86.6
Dabrafenib Melanoma, 2013	−10.2	49
Erlotinib First line NSCLC, 2013	−5.2	73.1
Nab−Paclitaxel, Adenocarcinoma Pancreatic , 2013	−2	76.5

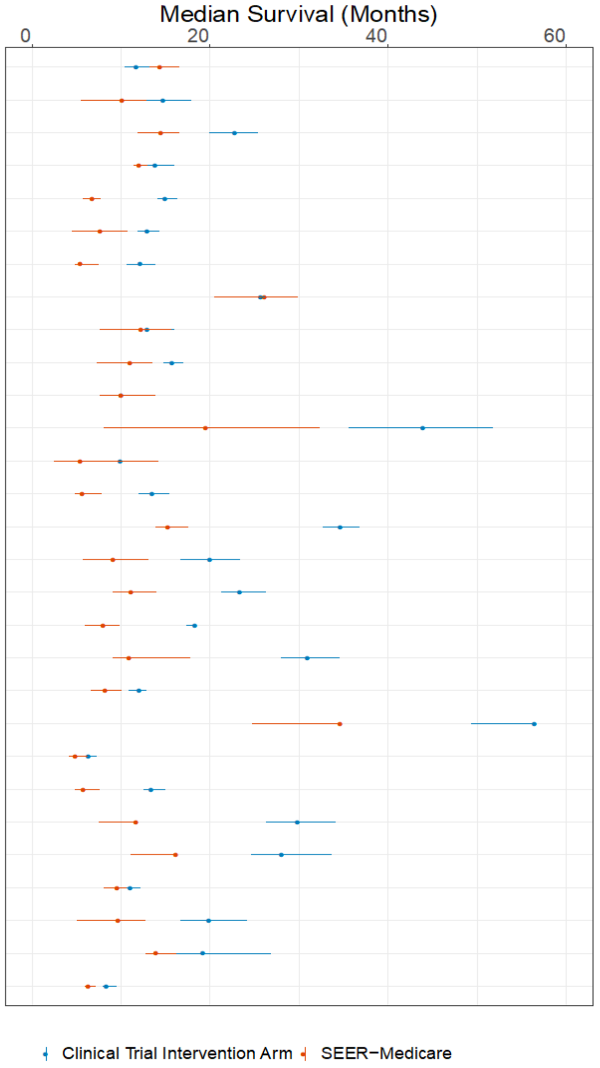

*Upper confidence bound not reached, SEER−Medicare (CCI 0−1)

© 2021 Green AK et al. *JAMA Network Open.*

eTable 1. SEER-Medicare Inclusion Criteria for Cohort Selection

Drug	Approval Year	FDA Approved Indication	SEER-Medicare Population Inclusion Criteria	Pivotal Cancer Clinical Trial
Pemetrexed, First Line NSCLC	2008	Pemetrexed for the initial treatment of patients with locally-advanced or metastatic non-squamous, non-small cell lung cancer in combination with cisplatin	Non-squamous, non-small cell lung cancer<br>Stage 4 at diagnosis<br>Stage 3 at diagnosis with no surgery claim within 365 days of the minimum date of claim for pemetrexed (J9305)<br><br>At least one claim for cisplatin (J9060) within 30 days of claim for pemetrexed (J9305)<br><br>No prior claim for other intravenous systemic therapy (J9000-J9998), erlotinib, gefitnib, afatinib, crizotinib, alectinib, or ceritinib from date of diagnosis to minimum claim date for pemetrexed (J9305)	https://ascopubs.org/doi/full/10.1200/JCO.2007.15.0375?url_ver=Z39.88-2003&rfr_id=ori:rid:crossref.org&rfr_dat=cr_pub%3dpubmed
Everolimus, RCC	2009	Everolimus for the treatment of advanced renal cell carcinoma after failure of treatment with sunitinib or sorafenib	Renal cell carcinoma<br>Stage 4 at diagnosis<br><br>Prior claim for sunitinib or sorafenib between date of diagnosis and minimum claim date for everolimus	RECORD-1<br>http://www.thelancet.com/retrieve/pii/S0140673608610399<br><br>https://onlinelibrary.wiley.com/doi/full/10.1002/cncr.25219

© 2021 Green AK et al. *JAMA Network Open*.

Drug	Approval Year	FDA Approved Indication	SEER-Medicare Population Inclusion Criteria	Pivotal Cancer Clinical Trial
Pazopanib, RCC	2009	Pazopanib for the treatment of patients with advanced renal cell carcinoma	Renal cell carcinoma<br><br>Stage 3 at diagnosis with no prior surgery within 365 days of the minimum claim date for pazopanib<br><br>Stage 4 at diagnosis<br><br>No prior claim for sunitinib, sorafenib, everolimus, or intravenous systemic therapy (J9000-J9998) between date of diagnosis and minimum claim date for pazopanib	https://ascopubs.org/doi/full/10.1200/JCO.2009.23.9764?url_ver=Z39.88-2003&rfr_id=ori:rid:crossref.org&rfr_dat=cr_pub%3dpubmed
Pemetrexed, Maintenance NSCLC	2009	Pemetrexed as maintenance treatment of patients with locally advanced or metastatic, non-squamous non-small cell lung cancer whose disease has not progressed after 4 cycles of platinum-based first-line chemotherapy, as a single agent	Non-squamous, non-small cell lung cancer<br>Stage 4 at diagnosis<br>Stage 3 at diagnosis with no surgery claim within 365 days of the minimum date of claim for pemetrexed (J9305)<br><br>Prior concurrent platinum therapy as defined by a claim for cisplatin (J9060) or carboplatin (J9045) plus pemetrexed within 30 days of each other AND claim for pemetrexed (J9305) alone within 60 days after platinum (cisplatin or carboplatin) plus pemetrexed (J9305)	PARAMOUNT<br>http://citeseerx.ist.psu.edu/viewdoc/download?doi=10.1.1.1007.6831&rep=rep1&type=pdf

© 2021 Green AK et al. *JAMA Network Open.*

Drug	Approval Year	FDA Approved Indication	SEER-Medicare Population Inclusion Criteria	Pivotal Cancer Clinical Trial
			No prior claim for other intravenous systemic therapy (J9000-J9998), erlotinib, gefitinib, afatinib, crizotinib, alectinib, or ceritinib from date of diagnosis to minimum claim date for [carboplatin or cisplatin] or pemetrexed (J9305)	
Cabazitaxel, mCRPC	2010	Cabazitaxel in combination with prednisone for the treatment of patients with hormone-refractory metastatic prostate cancer previously treated with a docetaxel-containing regimen.	Prostate adenocarcinoma<br>Stage 4 at diagnosis<br>No prior claim for mitoxantrone (J9293)<br>Prior claim for docetaxel (J9170, J9171) from date of diagnosis to minimum date of claim for cabazitaxel (J9043)	TROPIC https://www.thelancet.com/journals/lancet/article/PIIS0140-6736(10)61389-X/fulltext
Erlotinib, maintenance NSCLC	2010	Erlotinib as maintenance treatment of patients with locally advanced or metastatic non-small cell lung cancer whose disease has not progressed after four cycles of platinum-based first-line chemotherapy	Non-small cell lung cancer<br>Stage 4 at diagnosis<br><br>Stage 3 at diagnosis with no surgery claim within 365 days of the minimum date of claim for cisplatin (J9060) or carboplatin (J9045)<br><br>Prior platinum chemotherapy as defined by a claim for cisplatin (J9060) or carboplatin (J9045) from date of diagnosis to minimum date of claim for erlotinib	SATURN https://www.sciencedirect.com/science/article/pii/S1470204510701121?via%3Dihub

© 2021 Green AK et al. *JAMA Network Open.*

Drug	Approval Year	FDA Approved Indication	SEER-Medicare Population Inclusion Criteria	Pivotal Cancer Clinical Trial
			Claim for erlotinib within 60 days of maximum claim for cisplatin (J9060) or carboplatin (J9045)	
Eribulin, breast cancer	2010	Treatment of patients with metastatic breast cancer who have previously received an anthracycline and a taxane in either the adjuvant or metastatic setting, and at least two chemotherapeutic regimens for the treatment of metastatic disease	Breast cancer<br>Stage 4 at diagnosis<br>Previous claim for [taxane – paclitaxel (J9267, J9265) OR docetaxel (J9170, J9171) OR nab-paclitaxel (J9264)] AND [anthracycline – doxorubicin (J9000), liposomal doxorubicin (J9001, J9002)] from date of diagnosis to date of claim for eribulin (J9179)	EMBRACE<br>https://www.thelancet.com/journals/lancet/article/PIIS0140-6736(11)60070-6/fulltext
Trastuzumab, gastric cancer	2010	In combination with chemotherapy for first-line treatment of HER2-positive advanced gastric or gastro-oesophageal junction cancer.	Adenocarcinoma of the stomach or gastro-esophageal junction<br>Stage 3 at diagnosis with no surgery claim within 365 days of the minimum date of claim for trastuzumab (J9355)<br>No prior claim for intravenous systemic therapy (J9000-J9998) or capecitabine (J8520) between date of diagnosis and minimum claim date for trastuzumab (J9355)<br>At least one claim for [capecitabine (J8520, J8521) OR fluorouracil (J9190)] AND cisplatin (J9060, J9062) within 30 days of a claim for trastuzumab (J9355)	ToGA Trial<br>https://www.thelancet.com/journals/lancet/article/PIIS0140-6736(10)61121-X/fulltext

© 2021 Green AK et al. *JAMA Network Open*.

Drug	Approval Year	FDA Approved Indication	SEER-Medicare Population Inclusion Criteria	Pivotal Cancer Clinical Trial
Sipuleucel-T, CRPC	2010	Indicated for the treatment of asymptomatic or minimally symptomatic metastatic castrate resistant (hormone refractory) prostate cancer.	Prostate adenocarcinoma<br>Stage 4 at diagnosis	IMPACT<br>https://www.nejm.org/doi/full/10.1056/NEJMoa1001294
Everolimus, pancreatic NET	2011	Everolimus for the treatment of progressive neuroendocrine tumors of pancreatic origin that is locally advanced or metastatic. The safety and effectiveness in the treatment of patients with carcinoid tumors has not been established.	Pancreatic neuroendocrine tumor<br>Stage 4 at diagnosis<br>Stage 3 at diagnosis with no surgery claim within 365 days of the minimum date of claim for everolimus	RADIANT-3<br>https://ascopubs.org/doi/full/10.1200/JCO.2016.68.0702?url_ver=Z39.88-2003&rfr_id=ori:rid:crossref.org&rfr_dat=cr_pub%3dpubmed<br>https://www.nejm.org/doi/10.1056/NEJMoa1009290
Abiraterone, CRPC, prior docetaxel	2011	Abiraterone in combination with prednisone for the treatment of patients with metastatic castration-resistant prostate cancer who have received prior chemotherapy containing docetaxel	Prostate adenocarcinoma<br>Stage 4 at diagnosis<br>Prior claim for docetaxel (J9170, J9171) from date of diagnosis to minimum date of claim abiraterone	COU-AA-301<br>https://www.nejm.org/doi/10.1056/NEJMoa1014618?url_ver=Z39.88-2003&rfr_id=ori:rid:crossref.org&rfr_dat=cr_pub%3dwww.ncbi.nlm.nih.gov<br><br>http://www.thelancet.com/retrieve/pii/S1470204512703790
Vemurafenib, melanoma	2011	Treatment-naive *BRAF* V600 mutation–positive metastatic melanoma	Melanoma (cutaneous)<br>Stage 4 at diagnosis<br><br>Stage 3 at diagnosis with no surgery claim within 365 days of the	BRIM-3<br>https://www.ncbi.nlm.nih.gov/pmc/articles/PMC5834156/

© 2021 Green AK et al. *JAMA Network Open*.

Drug	Approval Year	FDA Approved Indication	SEER-Medicare Population Inclusion Criteria	Pivotal Cancer Clinical Trial
			minimum date of claim for vemurafenib<br><br>No prior claim for dacarbazine (J9130), temozolomide (J9328), carboplatin (J9045), or interleukin-2 (00.15)	https://www.nejm.org/doi/full/10.1056/NEJMoa1103782
Cetuximab, head and neck cancer	2011	Use in combination with platinum-based therapy plus fluorouracil (5-FU) for the first-line treatment of patients with recurrent locoregional disease or metastatic squamous cell carcinoma of the head and neck	Squamous cell carcinoma of the head and neck<br>Stage 4 at diagnosis<br>At least one claim for [Cisplatin (J9060) OR carboplatin (J9045)] AND fluorouracil (J9190) within 30 days of a claim for cetuximab (J9055)<br>Exclude nasopharyngeal cancers<br>No prior claim for intravenous systemic therapy (J9000-J9998) between date of diagnosis and minimum claim date for cetuximab (J9055)	EXTREME<br>https://www.nejm.org/doi/full/10.1056/NEJMoa0802656
Ipilimumab, melanoma	2011	Unresectable or metastatic melanoma who had received at least one prior systemic treatment	Melanoma (cutaneous)<br>Stage 4 at diagnosis<br>Stage 3 at diagnosis with no surgery claim within 365 days of the minimum date of claim for ipilimumab (J9228)<br>Previous claim for at least one of the following from date of diagnosis to minimum date of claim for ipilimumab (J9228): dacarbazine	https://www.nejm.org/doi/10.1056/NEJMoa1003466?url_ver=Z39.88-2003&rfr_id=ori:rid:crossref.org&rfr_dat=cr_pub%3dwww.ncbi.nlm.nih.gov

© 2021 Green AK et al. *JAMA Network Open.*

Drug	Approval Year	FDA Approved Indication	SEER-Medicare Population Inclusion Criteria	Pivotal Cancer Clinical Trial
			(J9130), temozolomide (J9328), carboplatin (J9045), or interleukin-2<br>Exclude ocular melanoma	
Everolimus, Breast	2012	Everolimus for the treatment of postmenopausal women with advanced hormone receptor-positive, HER2-negative breast cancer in combination with exemestane after failure of treatment with letrozole or anastrozole.	Breast ductal or lobular carcinoma<br>Stage 4 at diagnosis<br>Prior claim for anastrozole and/or letrozole<br>At least one claim for exemestane within 30 days of a claim for everolimus	BOLERO-2<br>https://www.nejm.org/doi/full/10.1056/nejmoa1109653
Axitinib, RCC	2012	Axitinib for the treatment of advanced renal cell carcinoma after failure of one prior systemic therapy.	Renal cell carcinoma<br>Stage 4 at diagnosis<br>Prior claim for sunitinib, bevacizumab (J9035) plus interferon-alpha (J9213), or temsirolimus (J9330), or interleukin-2 (00.15) between date of diagnosis and minimum claim date for axitinib	AXIS<br>https://www.thelancet.com/journals/lanonc/article/PIIS1470-2045(13)70093-7/fulltext<br><br>https://www.thelancet.com/journals/lancet/article/PIIS0140-6736(11)61613-9/fulltext
Nab-Paclitaxel, NSCLC	2012	Nab-Paclitaxel for treatment of locally advanced or metastatic non-small cell lung cancer, as first-line treatment in combination with carboplatin, in patients who are not candidates for curative surgery or radiation therapy	Non-small cell lung cancer<br><br>Stage 4 at diagnosis<br><br>Stage 3 at diagnosis with no prior surgery within 365 days of the minimum claim date for nab-paclitaxel (J9264), or no prior radiation claims lasting greater than 3 weeks from date of diagnosis and	http://ascopubs.org/doi/full/10.1200/JCO.2011.39.5848?url_ver=Z39.88-2003&rfr_id=ori:rid:crossref.org&rfr_dat=cr_pub%3dpubmed

© 2021 Green AK et al. *JAMA Network Open*.

Drug	Approval Year	FDA Approved Indication	SEER-Medicare Population Inclusion Criteria	Pivotal Cancer Clinical Trial
			minimum claim date for nab-paclitaxel (J9264)<br><br>No prior claim for intravenous systemic therapy (J9000-J9998), erlotinib, gefitnib, afatinib, crizotinib, alectinib, or ceritinib between date of diagnosis to minimum claim date for nab-paclitaxel (J9264)<br><br>At least one claim for carboplatin within 30 days of a claim for nab-paclitaxel (J9264)	
Enzalutamide, CRPC	2012	Enzalutamide for the treatment of patients with metastatic castration-resistant prostate cancer who have previously received docetaxel	Prostate adenocarcinoma<br>Stage 4 at diagnosis<br>Prior claim for docetaxel (J9170, J9171) from date of diagnosis to minimum date of claim for enzalutamide	AFFIRM<br>https://www.nejm.org/doi/10.1056/NEJMoa1207506?url_ver=Z39.88-2003&rfr_id=ori:rid:crossref.org&rfr_dat=cr_pub%3dwww.ncbi.nlm.nih.gov
Abiraterone, CRPC	2012	Abiraterone in combination with prednisone for the treatment of patients with metastatic castration-resistant prostate cancer	Prostate adenocarcinoma<br>Stage 4 at diagnosis	COU-AA-302<br>https://www.thelancet.com/journals/lanonc/article/PIIS1470-2045(14)71205-7/fulltext
Regorafenib, Colorectal cancer	2012	Metastatic colorectal cancer who have been previously treated with fluoropyrimidine-, oxaliplatin-, and irinotecan-	Colon or rectal cancer<br>Stage 4 at diagnosis<br>Prior claim for a fluoropyrimidine [fluorouracil (J9190) OR capecitabine J8520)], oxaliplatin (J9263),	CORRECT<br>https://www.thelancet.com/journals/lancet/article/PIIS0140-6736(12)61900-X/fulltext

© 2021 Green AK et al. *JAMA Network Open.*

**Drug**	**Approval Year**	**FDA Approved Indication**	**SEER-Medicare Population Inclusion Criteria**	**Pivotal Cancer Clinical Trial**
		based chemotherapy, an anti-VEGF therapy, and, if KRAS wild type, an anti-EGFR therapy.	irinotecan (J9206), AND bevacizumab (J9035) since date of diagnosis and prior to claim for regorafenib	
Cetuximab, colorectal cancer	2012	First-line treatment of patients with KRAS mutation-negative (commonly known as KRAS wild-type), epidermal growth factor receptor (EGFR)-expressing metastatic colorectal cancer (mCRC) as determined by FDA-approved tests for this use	Colon or rectal adenocarcinoma<br>Stage 4 at diagnosis<br>At least one claim for irinotecan (J9206) AND fluorouracil (J9190) within 30 days of a claim for cetuximab (J9055)<br>No prior claim for intravenous systemic therapy (J9000-J9998) or capecitabine (J8520) between date of diagnosis and minimum claim date for cetuximab (J9055)	CRYSTAL<br>https://www.nejm.org/doi/full/10.1056/NEJMoa0805019<br><br>https://ascopubs.org/doi/full/10.1200/JCO.2010.33.5091?url_ver=Z39.88-2003&rfr_id=ori:rid:crossref.org&rfr_dat=cr_pub%3dpubmed
Pertuzumab, breast cancer	2012	In combination with Herceptin (trastuzumab) and docetaxel chemotherapy for the treatment of people with HER2-positive metastatic breast cancer (mBC) who have not received prior anti-HER2 therapy or chemotherapy for metastatic disease.	Breast cancer<br>Stage IV at diagnosis<br>No prior claim for intravenous systemic therapy (J9000-J9998) or lapatinib or capecitabine (J8520) between date of diagnosis and minimum claim date for pertuzumab (J9306)<br>At least one claim for trastuzumab (J9355) and docetaxel (J9170, J9171) within 30 days of a claim for pertuzumab (J9306)	CLEOPATRA<br>https://www.nejm.org/doi/full/10.1056/NEJMoa1413513
Ziv-Aflibercept	2012	Treatment of metastatic colorectal cancer (mCRC) that is resistant to or has	Colon or rectal adenocarcinoma<br>Stage 4 at diagnosis	VELOUR<br>http://ascopubs.org/doi/full/10.1200/JCO.2012.42.8201?ur

© 2021 Green AK et al. *JAMA Network Open.*

Drug	Approval Year	FDA Approved Indication	SEER-Medicare Population Inclusion Criteria	Pivotal Cancer Clinical Trial
colorectal cancer		progressed following an oxaliplatin-containing regimen	Prior claim for oxaliplatin (J9263) from date of diagnosis to minimum date of claim for ziv-aflibercept (J9400)<br>At least one claim for irinotecan (J9206) AND fluorouracil (J9190) within 30 days of a claim for ziv-aflibercept (J9400)<br>Exclude if prior claim for irinotecan from date of diagnosis to date of claim for ziv-aflibercept (J9400)	l_ver=Z39.88-2003&rfr_id=ori:rid:crossref.org&rfr_dat=cr_pub%3dpubmed
Ado-Trastuzumab, breast cancer	2013	For patients with HER2-positive, late-stage (metastatic) breast cancer.	Breast cancer<br>Stage 4 at diagnosis<br>Stage 3 at diagnosis with no surgery claim within 365 days of the minimum date for ado-trastuzumab (J9354)<br>Prior claim for trastuzumab (J9355) AND [paclitaxel (J9265, J9267), docetaxel (J9170, J9171) OR nab-paclitaxel (J9264)]<br>No prior treatment with lapatinib or capecitabine (J8520, J8521)	EMILIA<br>https://www.nejm.org/doi/10.1056/NEJMoa1209124?url_ver=Z39.88-2003&rfr_id=ori:rid:crossref.org&rfr_dat=cr_pub%3dwww.ncbi.nlm.nih.gov<br><br>https://www.thelancet.com/journals/lanonc/article/PIIS1470-2045(17)30312-1/fulltext
Dabrafenib, Melanoma	2013	Indicated for the treatment of patients with unresectable or metastatic melanoma with BRAF V600E mutation as detected by an FDA-approved test.	Melanoma, cutaneous<br>Stage 4 at diagnosis<br>Stage 3 at diagnosis with no surgery claim within 365 days of the minimum date of claim for dabrafenib	BREAK-3<br>https://www.sciencedirect.com/science/article/pii/S014067361260868X?via%3Dihub

© 2021 Green AK et al. *JAMA Network Open*.

Drug	Approval Year	FDA Approved Indication	SEER-Medicare Population Inclusion Criteria	Pivotal Cancer Clinical Trial
			No prior claim for intravenous systemic therapy (J9000-J9998), except interleukin-2, between date of diagnosis and date of claim for dabrafenib	
Nab-Paclitaxel, pancreatic	2013	Nab-Paclitaxel for metastatic adenocarcinoma of the pancreas as first line treatment, in combination with gemcitabine	Pancreatic adenocarcinoma<br>Stage 4 at diagnosis<br>Claim for gemcitabine (J9201) within 30 days for claim for nab-paclitaxel (J9264) and no prior claim for other intravenous systemic therapy (J9000-J9998) or capecitabine (J8520) from date of diagnosis to minimum claim date for nab-paclitaxel (J9264)	https://www.nejm.org/doi/full/10.1056/NEJMoa1304369
Afatinib, NSCLC	2013	For patients with late stage (metastatic) non-small cell lung<br>cancer (NSCLC) whose tumors express specific types of<br>epidermal growth factor receptor (EGFR) gene mutations, as<br>detected by an FDA-approved test. Data from LUX-Lung 3 trial of afatinib vs. cisplatin/pemetrexed.	Non-small cell lung cancer<br>Stage 4 at diagnosis<br>Stage 3 at diagnosis with no surgery or radiation claim within 365 days of the minimum date of claim for afatinib<br><br>No prior claim for other intravenous systemic therapy (J9000-J9998), erlotinib, gefitinib, crizotinib, alectinib, or ceritinib from date of diagnosis to minimum claim date for afitinib	LUX Lung 3, LUX Lung 6<br>https://www.thelancet.com/journals/lanonc/article/PIIS1470-2045(14)71173-8/fulltext<br><br>https://ascopubs.org/doi/abs/10.1200/jco.2012.30.18_suppl.lba7500
Erlotinib, NSCLC	2013	The treatment of patients with metastatic non-small cell lung cancer whose tumors have epidermal growth factor	Non-small cell lung cancer<br>Stage 4 at diagnosis	EURTAC<br>https://www.thelancet.com/action/showPdf?pii=S1470-2045%2811%2970393-X

© 2021 Green AK et al. *JAMA Network Open*.

Drug	Approval Year	FDA Approved Indication	SEER-Medicare Population Inclusion Criteria	Pivotal Cancer Clinical Trial
		receptor (EGFR) exon 19 deletions or exon 21 (L858R) substitution mutations as detected by an FDA-approved test receiving first-line, maintenance, or second or greater line treatment after progression following at least one prior chemotherapy regimen.	Stage 3 at diagnosis with no surgery or radiation claim within 365 days of the minimum date of claim for erlotinib<br><br>No prior claim for other intravenous systemic therapy (J9000-J9998), afatinib, gefitinib, crizotinib, alectinib, or ceritinib from date of diagnosis to minimum claim date for erlotinib	
Bevacizumab, Colorectal Cancer	2013	Metastatic colorectal cancer, in combination with fluoropyrimidine irinotecan- or fluoropyrimidine-oxaliplatin-based chemotherapy for second-line treatment in patients who have progressed on a first-line Avastin-containing regimen.	Colon or rectal cancer<br>Stage 4 at diagnosis<br>Prior claim for bevacizumab (J9035) AND [fluorouracil (J9190) OR capecitabine (J8520, J8521)] AND [oxaliplatin (J9263) OR irinotecan (J9206)]<br>At least one claim for [irinotecan (J9206) OR oxaliplatin (J9263)] AND [capecitabine (J8520, J8521) OR fluorouracil (J9190)] within 30 days of a claim for bevacizumab (J9035).	ML18147 Study<br>https://www.thelancet.com/action/showPdf?pii=S1470-2045%2812%2970477-1

CRPC=Castrate Resistant Prostate Cancer; NET=Neuroendocrine tumor; NSCLC=Non-Small Cell Lung Cancer; RCC=Renal Cell Carcinoma

© 2021 Green AK et al. *JAMA Network Open.*

eTable 2. Cycle Estimates for Intravenous Cancer Drugs Used to Calculate Duration of Therapy and/or Single Cycles

Drug and Indication	FDA Label Dosing Frequency	Cycle Estimate in SEER-Medicare
Pemetrexed, first line NSCLC	Recommended dose is 500 mg/m^2 IV on Day 1 of each 21-day cycle in combination with cisplatin 75 mg/m^2 IV beginning 30 minutes after ALIMTA administration.	One claim every 21 days
Nab-Paclitaxel, NSCLC	Recommended dose is 100 mg/m^2 over 30 minutes on Days 1, 8, and 15 of each 21-day cycle; administer carboplatin on Day 1 of each 21-day cycle immediately after ABRAXANE.	At least two claims for nab-paclitaxel within 21 days
Nab-Paclitaxel, Pancreatic Adenocarcinoma	Recommended dose is 125 mg/m^2 IV over 30-40 minutes on Days 1, 8 and 15 of each 28-day cycle; administer gemcitabine on Days 1, 8 and 15 of each 28-day cycle immediately after ABRAXANE.	At least two claims for nab-paclitaxel within a 28 day period
Pemetrexed, maintenance NSCLC	Recommended dose is 500 mg/m^2 IV on Day 1 of each 21-day cycle.	One claim every 21 days
Cabazitaxel, CRPC	20 mg/m^2 administered every three weeks as a one-hour intravenous infusion in combination with oral prednisone 10 mg administered daily throughout JEVTANA treatment.	One claim every 21 days
Eribulin, Breast cancer	Administer 1.4 mg/m^2 intravenously over 2 to 5 minutes on Days 1 and 8 of a 21-day cycle.	Two claims every 21 days
Trastuzumab, Gastric cancer	Initial dose of 8 mg/kg over 90 minutes IV infusion, followed by 6 mg/kg over 30 to 90 minutes IV infusion every 3 weeks.	One claim every 21 days
Cetuximab, Head and Neck	Administer 400 mg/m^2 initial dose as a 120-minute intravenous infusion followed by 250 mg/m^2 weekly infused over 60 minutes.	Three claims every 21 days
Cetuximab, Colorectal cancer	Administer 400 mg/m^2 initial dose as a 120-minute intravenous infusion followed by 250 mg/m^2 weekly infused over 60 minutes.	Three claims every 21 days

© 2021 Green AK et al. *JAMA Network Open.*

**Drug and Indication**	**FDA Label Dosing Frequency**	**Cycle Estimate in SEER-Medicare**
Ipilimumab, Melanoma	3 mg/kg administered intravenously over 90 minutes every 3 weeks for a total of 4 doses.	One claim every 21 days
Pertuzumab, Breast cancer	The initial dose is 840 mg administered as a 60-minute intravenous infusion, followed every 3 weeks thereafter by 420 mg administered as a 30 to 60 minute intravenous infusion.	One claim every 21 days
Ziv-Aflibercept, Colorectal cancer	4 mg/kg as an intravenous infusion over 1 hour every 2 weeks	One claim every 14 days
Ado-trastuzumab, Breast cancer	The recommended dose of KADCYLA is 3.6 mg/kg given as an intravenous infusion every 3 weeks (21-day cycle)	One claim every 21 days
Bevacizumab, Colorectal	5 mg/kg IV every 2 weeks with bolus-IFL; 10 mg/kg IV every 2 weeks with FOLFOX4	One claim every 14 days

CRPC=Castrate Resistant Prostate Cancer; NSCLC=Non-small cell lung cancer

© 2021 Green AK et al. *JAMA Network Open*.

eTable 3. Median Cancer-Specific Survival and Percent Deaths Due to Cancer Among SEER-Medicare Patients, Censored 12/31/2015

Drug and Indication	Cancer-specific survival, median in months	Percent deaths attributed to cancer (%)
Nab-Paclitaxel, NSCLC	9.4	89.1
Nab-Paclitaxel, Pancreatic Adenocarcinoma	6.7	92.7
Everolimus, Breast	11.7	81.6
Erlotinib, NSCLC	7.0	90.6
Pemetrexed, Maintenance NSCLC	16.2	89.6
Everolimus, RCC	10.9	86.1
Pemetrexed, First Line NSCLC	15.7	88.1
Pazopanib, RCC	13.4	90.9
Axitinib, RCC	8.9	90.0
Enzalutamide, CRPC	8.7	92.1
Abiraterone, No Prior Docetaxel CRPC	18.0	78.5
Abiraterone, Prior Docetaxel CRPC	11.1	90.0
Everolimus, Pancreatic NET	20.9	86.7
Cabazitaxel, CRPC	7.0	89.1
Ipilimumab, Melanoma	5.3	91.3
Regorafenib, Colorectal	4.7	90.5
Eribulin, Breast	7.9	95.8
Vemurafenib, Melanoma	5.3	92.4
Ziv-Aflibercept, Colorectal	5.3	90.3
Cetuximab, Colorectal	11.2	89.9
Pertuzumab, Breast	Not Reached	81.8
Cetuximab, Head and Neck	11.0	82.7

© 2021 Green AK et al. *JAMA Network Open.*

Drug and Indication	Cancer-specific survival, median in months	Percent deaths attributed to cancer (%)
Trastuzumab, Gastric	15.0	88.2
Afatinib, NSCLC	12.0	78.9
Ado trastuzumab, Breast	7.5	100.0
Dabrafenib, Melanoma	9.8	85.2
Erlotinib, First line NSCLC	13.6	90.8
Sipuleucel-T, CRPC	28.2	84.2
Bevacizumab, Colorectal	9.7	93.6

CRPC=Castrate Resistant Prostate Cancer; NET=Neuroendocrine tumor; NSCLC=Non-Small Cell Lung Cancer; RCC=Renal Cell Carcinoma

© 2021 Green AK et al. *JAMA Network Open*.

eTable 4. Age-Specific Median Overall Survival or Hazard Ratios for Clinical Trial Intervention Arms

**Drug Indication**	**Age group**	**Trial Overall Survival, months**	**Hazard Ratio (95% CI)**
Enzalutamide CRPC	≥ 65 years	18.4	0.63 (0.46-0.87)
	<65 years	Not reached	0.63 (0.51-0.78)
	All	18.4	0.63 (0.53-0.75)
Abiraterone CRPC, prior docetaxel	≥ 65 years	16.2	0.76 (0.63-0.90)
	<65 years	15.0	0.69 (0.53-0.91)
	≥ 75 years	15.6	0.64 (0.48-0.85)
	All	15.8	0.74 (0.64-0.86)
Abiraterone CRPC, no prior docetaxel	≥ 65 years	34.5	0.78 (0.59-1.03)
	<65 years	34.7	0.81 (0.69-0.96)
	All	34.7	0.81 (0.70-0.93)
Nab-Paclitaxel, NSCLC	≥ 70 years	19.9	0.58, significant
	< 70 years	11.4	0.99, NS
	All	12.1	0.92 (0.80-1.07)
Cetuximab, Head and Neck	≥ 65 years	9.1	1.07 (0.65-1.77)
	<65 years	10.5	0.74 (0.59-0.94)
	All	10.1	0.80 (0.64-0.99)
Afatinib, NSCLC	≥ 65 years	27.6	0.94 (0.58-1.51)
	<65 years	29.4	0.59 (0.33-1.05)
	All	28.2	0.88 (0.66-1.17)

*NS=Non-significant hazard ratio for overall survival as reported in trial publication
CRPC=Castrate Resistant Prostate Cancer; NSCLC=Non-Small Cell Lung Cancer

© 2021 Green AK et al. *JAMA Network Open*.

© 2021 Green AK et al. *JAMA Network Open.*
